# A Medico-Legal Treatise on Homicide by External Violence

**Published:** 1838-01

**Authors:** 


### Art. XIII.

*A Medico-legal Treatise on Homicide by external Violence; with an Account of the Circumstances which modify the Medico-legal Characters of Injuries and exculpatory Pleas.* By Alexander Watson, Fellow of the Royal College of Surgeons, Edinburgh, and one of the Surgeons to the Royal Infirmary, &c. &c. &c.—*Edinburgh,* 1837. 8vo. pp. 355.

Mr. Watson, the author of the work before us, has long been known to the profession as a contributor of articles on Medical Jurisprudence to a respected contemporary Journal. That he has been a most industrious labourer in this department of science, the treatise which he has now published sufficiently shews. But other circumstances have concurred to place him in a most favorable situation for collecting and recording facts. There are probably few in Edinburgh, and we may confidently say there is not one medical practitioner in London, who can boast of having been consulted judicially in one-half of the number of cases in which we find, by this volume, Mr. Watson has been personally engaged. We rejoice to perceive that this golden opportunity of collecting interesting medico-legal facts has fallen to so shrewd an observer, and, generally speaking, so cautious a reasoner. The work, we are assured, will be welcomed not only by all professed medical jurists, but by those who have not yet been led to consider medico-legal knowledge as of great importance to a practising surgeon. We invite the attention of the latter class in particular to this volume. The great bulk of it refers to the duties of a *surgeon;* and its perusal will suffice to shew, not only that the kind of information which it contains is not to be obtained either from an attendance on surgical lectures or from a perusal of the works of surgical writers, but that, without this knowledge, the personal reputation of the practitioner is endangered, and the course of public justice must be embarrassed.

Although Mr. Watson's work is especially devoted to a consideration of the subject of Homicide by "external violence," yet we find treated of, the varieties of homicide by asphyxia, spontaneous combustion, death by lightning, and infanticide. We do not object to the introduction of these accessory subjects; but the somewhat hasty manner in which they are dismissed rather tends to confuse than enlighten the reader. This, as we shall presently see, is particularly the case with the chapter on Infanticide; some of the most important questions connected with which are discussed in two or three pages. One striking feature in the volume is the number of cases which are distributed through it. Many of these have been derived from the author's personal experience, but with others, extracted from the works of Sir A. Cooper, Sir C. Bell, and Mr. Travers, most of our readers will be familiar. We are at a loss to conceive why some of the cases are introduced, since they occupy only a few lines, are unaccompanied with any comment, and merely illustrate facts which must be well known even to a tyro in the profession. Thus, the case of Dr. Hunter (p. 213,) is inserted in *three lines*, to shew that a man may die suddenly, while in a state of apparent health. Had there been fewer cases, and the comments of the author more frequent upon those which are inserted, they would, we think, have served the purpose of instruction better; for, after all, it is not so much their number as their individual value which really benefits the medico-legal student. We trust that this hint will not be lost in a second edition of the work; since, assuredly, the most instructive cases in the treatise are those in which the author has marked out the details, and exposed in clear language the reasons upon which his inferences were founded.

In defining the nature and objects of medical jurisprudence, after stating that "the duty" of the medical jurist is, by his science, in certain cases to assist judges and juries in the administration of justice, we find the following excellent remarks, which should be borne in mind by every practitioner who is called to act in a medico-legal capacity.

> "In the exercise of this duty, it is obvious that the medical jurist must neither adopt nor endeavour to support one side or other of any case, when his opinion is required on it, to the exclusion of a just regard to the opposite side. If he did so, the ends of justice could not be accomplished. Such a proceeding would be a degradation of his character, and a prostitution of his profession. When it has been attempted, conflicting opinions have been given by the medical witnesses on the different sides of the case, arising either from a slight distortion of the facts to suit their own particular views, or from ill-judged zeal to maintain the side of the party by whom they have been requested to appear. These medical men have mistaken the nature of the duty which they had to perform. In place of acting as judges on the points referred to them for decision, they have become the advocates and partisans of one side of the case; and, sometimes, what ought to have been an opinion on the facts of the case before them has assumed the controversial character of a speculative debate; and this to the astonishment of the judges and jury, the amusement of the lawyers, the injury of parties, the obstruction of justice, the ridicule and contempt of the public, and the degradation of the medical profession." (P. 7.)

These remarks, which are evidently the result of long observation on the general conduct of medical witnesses, are followed up by a very proper caution, too often neglected, namely, that a medical jurist should never give an opinion on a case without first ascertaining *all the facts* connected with it.

A large portion of the volume is occupied with the medico-legal history of Wounds. It is upon this subject that the writer has evidently had the largest experience, and has bestowed the greatest attention. Mr. Watson must excuse us if we consider his definition of a "wound" but little calculated to remove the vagueness, the existence of which among medico-legal reports of the present day he makes a formal matter of complaint. Thus, he includes fractures and dislocations among wounds; but he omits ruptures of the viscera, which, however, must fall under his acceptation of the term wound. Fortunately, the necessity for a strict understanding among medical witnesses in England on this point has now passed away. A recent statute (1 Victoria, c. 85,) has already provided a remedy for those defects in our criminal law, to which we called the attention of our readers in a late Number.

The first class of injuries treated of are those affecting the *nervous* system. These are very fully considered, and well illustrated by cases. The diagnosis of concussion, with its attendant symptoms, is well laid down. In speaking of the appearances occasionally met with in the cranium, the author does not omit to state the important medico-legal fact, "that concussion of the brain may prove fatal, without either fracture of the skull, effusion of blood within the cranium, or any other change being observed on dissection." (P. 35.) Cases are subjoined, which fully confirm the truth of this statement.

We next find a report of eight cases, to prove that a *blow of the fist* on the head may cause death, by producing concussion, effusion of blood, or both. The author attempts (but, in our opinion, not very successfully,) to determine whether, in these instances, the injury to the brain is caused by the blow or by the fall on the head, when the party is knocked down; a question frequently put to a medical witness, and the answer to which may have sometimes a material influence on the case of a prisoner. In several subjects which Mr. Watson examined, the chief clot of effused blood was on the side *opposite* to the external mark of injury which occasioned it. (P. 36.) Further,

"Although, in some cases of commotion, (concussion,) no change of structure can be observed in the brain after death, yet we cannot infer from this that lesion of the brain has not been produced. When death happens immediately or soon after the injury, time has not been allowed for subsequent changes to take place, which might have pointed out organic lesion on dissection. Hemorrhage, for example, may be suspended by the depressed state of the circulation which takes place, but afterwards occurs when reaction is established and produces compression of the brain. The same may be said of inflammation, which generally supervenes, if the patient recovers from the primary shock of the injury. Besides, the ultimate structure of the brain is so minute that some organic injuries of it may not be cognizable to our senses." (P. 45.)

An extravasation of blood, as a consequence of laceration of the brain, is often situated within the dura mater; rarely at the place where the blow has been inflicted, but most commonly at the base of the brain. Extravasated blood has been met with from violence, even when there was no external mark of injury upon the head. The question, whether the effusion of blood has arisen from violence or from intoxication and disease, is next examined. In the view of the author, the situation of the effusion may sometimes serve as a criterion of its origin. The effusion from violence is generally situated upon the *surface* of the brain, while that which is the consequence of diseased vessels is commonly found within its substance. Of course, a fact of this kind can only be taken as *presumptive* of the cause of extravasation. The coexistence of a fracture with effusion of blood on the surface of the organ would render it almost certain that violence must have been the cause. The medical jurist is advised to be careful how he receives the dying declarations of persons who have suffered from concussion, but who have recovered sufficiently before death to converse upon their condition and the circumstances under which they were violently treated. Such persons, it is well known, have, in general, "no recollection of what took place immediately previous to or after the injury;" and, therefore, their statements regarding events that preceded the blow should not be trusted, unless confirmed by other evidence. (P. 57.)

In speaking of Inflammation and Suppuration of the Brain, the author adduces the following singular case, to shew the insidiousness of this process, as well as the slight cause from which it may occasionally proceed.

"Case 41. William Watt, aged ten, had the birch end of a scavenger's broom thrust into his face two or three times, by one of his companions. By this he was so stunned, that he lay down and was carried home in a state of stupor. He afterwards complained of violent pain in the eyeball and forehead. Symptoms of inflammation and fever supervened, followed by coma, insensibility, and convulsions. These, by active treatment, subsided; suppuration took place in the orbit; but he again relapsed, and died seventeen days after the accident. On dissection, there was an opening in the orbitar plate of the frontal bone, which extended into the brain, and an effusion of lymph and pus at the base of the brain. The left ventricle contained three ounces of pus, and communicated with the wound in the orbit. There was also considerable softening and disorganization of the brain. A small portion was partially separated from the orbitar plate, and projected upwards." (P. 64.)

As injuries of the brain may, in the first instance, appear very slight, but at the same time may destroy life after the lapse of months, or even years, so should a medical jurist be cautious how he pronounces the individual to be out of danger. He must remember that, in these cases, there may be danger, though it is not apparent, owing to the absence of symptoms. We cannot join our author, however, in believing that any legal difficulty, of the nature imagined by him, would arise, owing to a prematurely expressed medical opinion on these occasions.

"It has often happened," he says, "that the individual, A., has assaulted another individual, B., and given him a blow upon the head. B. does not appear to be much the worse of[for] the injury, so that in a few days A. is taken to the police-court, or before the sitting magistrate, and is tried for the assault. After an interval of a few days or weeks, however, B. is taken ill with inflammation, and dies from the effects of the injury. A. is therefore again laid hold of, and indicted to stand trial for murder. By a law of the land, no person can be tried twice for the same crime; so that a difference of opinion has been given in cases of this kind." (P. 67.)

The author's hypothetical case appears to us to involve the assumption of an assault and a murder being the *same crime*. The plea of "*autrefois convict*" in England is very clearly laid down by Hale, Blackstone, and other eminent authorities. "A previous conviction can now only be pleaded in bar of any subsequent indictment for the *felony*, of which the defendant has previously been convicted." (*Archbold Plead. and Ev.* 80.) It is, therefore, absurd to suppose that a previous conviction for a misdemeanour will be a bar to a subsequent indictment for a capital felony.

There are some interesting remarks relative to Injuries of the Trunk and Limbs, causing death by probably sympathetic influence between the nerves and the brain. One point, which is generally omitted by medico-legal writers, is properly adverted to by Mr. Watson,—namely, that "injuries which would not have been fatal individually, sometimes prove so by their combination or multiplicity." (P. 76.) The witness should not lose sight of this fact, when he is asked by a coroner, magistrate, or barrister, which, of several wounds on a deceased person, was *mortal?* A complication of injuries may as surely destroy life as any single wound which traverses a vital part. In speaking of the cause of death in *burns*, the author quotes the very obscure language of Mr. Travers, in which that gentleman refers the fatal effects to what he terms "functional concussion" of the brain; a form of expression rather tending to confuse than to assist our understanding of the subject. The phenomena of spontaneous combustion are referred, in our opinion very properly, to preternatural combustibility of the body; the ignition always proceeding from some external cause.

The second division of the subject brings us to Injuries of the *Circulating* System, which often demand medico-legal investigation. The loss of from five to eight pounds of blood, according to Mr. Watson, is required to prove fatal in cases of adults; but, in order that death should take place by hemorrhage, it is by no means necessary that a large blood-vessel should be opened. A wound of any very vascular part may be equally fatal. Two interesting cases which occurred to the author, one of which we shall presently notice, fully bear out the correctness of this view. In them the vagina was the seat of injury.

The means for detecting spots of blood on linen and weapons are so imperfectly detailed, that, in our opinion, they had better have been omitted. The author is not correct in stating that "ammonia changes the colour of all other red dyes," (p. 92,) except the colouring matter of the blood. Alizarine, the red colouring principle of madder, one of the most common red dyes, is not, according to our observation, changed by ammonia.

The consideration of Death by Hemorrhage affords room for the introduction of numerous interesting cases, illustrating the various degrees of mischief to the circulating system under which a fatal result may ensue. We learn from some of these that the loss of a few ounces of blood from the heart, provided it cannot freely escape from the pericardium, may destroy life; that a rupture of the heart or its larger vessels may take place from a blow or a fall, without leaving any traces of fracture or injury externally; that a wound of the internal mammary artery, or of one of the intercostal arteries, may as effectually kill as if the aorta were the seat of mischief; facts, upon the important bearing of which in medico-legal practice it is needless for us to dwell.

The third division embraces Injuries of the *Respiratory* System. After a slight outline of the phenomena of death by asphyxia, the author passes to the consideration of its various forms. His view of the cause of death may be regarded as a kind of compromise between the exclusive opinions of Bichat and his opponents. He allows that life is extinguished owing to the noxious influence, as well as the diminished quantity of blood sent to the brain. We find nothing new under the head of "Suffocation;" but we are tempted to extract the following instance of "accidental strangulation," from the singular circumstances under which it occurred. It is

"The case of an old woman, a salt carrier, who, on her journey, rested her creel upon a wall: it fell over the wall, and, the belt of it being round her neck, she was strangled by the weight of the creel. The same happened to a fishwoman, near St. Andrew's, a few years ago." (P. 126.)

Throughout the volume we have had occasion to notice a general neglect, on the part of Mr. Watson, of the labours of British and continental medical jurists. In his preface he apologises for this, by ascribing it to the active toils of the practical part of his profession. Willing, as we are, to admit the reasonableness of this apology, it does not form, as we conceive, a justification for the diffusion of an erroneous opinion. An author, who is obliged to trust solely to his own observation, should be proportionally cautious in making generalizations, which may have an important practical influence. We are sorry to meet with a neglect of this proper caution in the statement respecting the effects of a ligature on the neck, "that ecchymosis could not be produced by tying a cord after death." (P. 131.) Had the author consulted the recent works of Divergie and Casper, he would have found that the statement, however true in reference to his own experience, was wholly refuted by the experiments and observations of these distinguished writers. In the section on Hanging, we find also several passages which, we are sure, would have been, under these circumstances, modified or omitted. At page 142 we meet with the following: "If, upon examination, ecchymosis can be observed at any part in the rope-mark, it must have been formed during life."

To the chapter on Drowning we find prefixed some observations on the "physical effects of water in relation to the human body." We must charitably suppose that these have been hastily put together, and printed without revision. It is impossible that Mr. Watson can really entertain the singular notions which we here find announced under the title of the "General Laws of Hydrostatics." He first speaks of "a *square inch* of lead being heavier than a *square inch* of water," (p. 144;) and we next meet with the startling position, that, "when a floating body is depressed or forced down below the surface of the water, its specific gravity is increased by the pressure or weight of the superincumbent fluid upon it." (ib.) We should like to be informed how the specific gravity of a hollow copper ball, for example, becomes increased by submersion; and whether, when sunk, it must not be kept down by a weight proportioned to the difference between its specific gravity and that of water? Mr. Watson seems entirely to forget that there is an upward as well as a downward pressure; and that, in all *floating* bodies,—bodies really *lighter* than water,—unless the cause of their buoyancy be removed, the upward pressure will overcome the weight of the superincumbent fluid, and prevent the body from remaining anywhere but on the surface. The author next refers to what he calls the point of equipoise or equilibrium. As he considers a knowledge of this to be important in its application to the human body, and that it "affords an explanation of some of the phenomena which occur in drowning," we shall here quote his remarks:

"If the floating body is depressed to a sufficient depth, its specific gravity will increase until it becomes equal to that of the water. When the body arrives at this point, it will neither ascend nor descend in the water, but will remain stationary. Hence this has been called the point of equipoise or equilibrium. Now, it will be evident that, if the body is raised in any degree above the point of equilibrium, its specific gravity being lessened by a diminution of pressure upon it, the body immediately rises to the surface, and floats. And when, on the contrary, the body is depressed below the point of equilibrium, the increased pressure, by causing an augmentation of specific gravity, makes the body sink with a velocity greater and greater as it descends." (P. 145.)

We must confess our surprise at finding such palpably erroneous statements in a work of this description. A *floating* body, unless the cause of buoyancy be artificially removed from it, can have no point of equipoise except that which is actually close to the surface of the water. Equilibrium is not given to a *floating* body by sinking it to a certain depth, nor can it remain for an instant at this supposed point of equipoise, unless the weight which actually depressed it still continues attached to it. Admitting, however, that Mr. Watson had here given us the true exposition of a natural law, we must have some reason for a body remaining stationary at this point of equipoise; as also for the fact of its rising to the surface, and floating, when it accidentally gets a little *above*, or sinking deeper and deeper, with increased velocity as it descends, when it falls a little *below* that point. What can cause a body to rise higher, or sink with increased velocity lower, than that level in which it is already in equilibrium with water? The author states that "these phenomena are easily demonstrated by experiment." We need hardly observe, that his views upon this branch of physics are wholly inapplicable to the phenomena of death by drowning.

The section on the effects of "excessive Intoxication" is full of valuable practical remarks, for which we must refer our readers to the work itself. We object to the term asphyxia applied to this condition; since it appears to us to be as much a real form of poisoning as in the case of the exhibition of opium or any other poison.

We are much pleased with the chapter on the Medico-legal circumstances which modify Homicide. We think the author has been misinformed respecting the case of the fisherman, who was shot by Captain Moir, in 1830. If we mistake not, the deceased was wounded in the arm, not in the thigh; and died from tetanus, not from erysipelas. The cases in this part of the work are, however, given with great care and precision, and are highly interesting to the medical jurist. One of these, in illustration of the subject of *malum regimen*, after the receipt of severe injuries, we here present to our readers:

"J. Bell was tried at Edinburgh, November 14, 1836, for the murder of John Kerr, aged nineteen, by having struck him a severe blow on the forehead with a hoe, on the 20th July preceding, whereby he died on the 28th of the same month. Kerr was admitted into the Royal Infirmary, under the care of Professor Lizars, on the day after the injury, when he was able to walk to his bed, and did not seem to be very ill. The post-mortem examination, at which I was present, was conducted by Mr. Fergusson and Dr. W. Home, who drew up the official report on the case.

"About an inch above the right orbit, and close to the mesial line, there was a circular "compound, comminuted, and depressed fracture of the middle part of the frontal bone;" the depression having the appearance of a shallow cup, of about half an inch in depth. The *external* wound of the scalp was like three incisions meeting at one of their extremities; the pericranium was in a sloughy state; the wound was in a state of suppuration. Internally, there was purulent matter between the broken cranium and dura mater; some spiculæ of bone attached to the dura mater, several of which had penetrated through it into the brain. There was some purulent matter and lymph below the dura mater, and softening with abscess in the substance of the brain, about an inch deep at the seat of the injury. The medullary substance of the brain was inflamed, and had a yellow appearance, to the extent of about an inch and a half around the abscess.

"A simple fracture extended from the circular depressed fracture into the orbit; another, about an inch upwards, to the crown of the head. No operation had been performed to remove the depressed portions of bone." (P. 236.)

Upon this case the author makes the following comments:

"This young man was in good health when he received the injury, and evidently died from inflammation of the brain. When he came to the infirmary, the symptoms of inflammation of the brain did not seem to have come on; nevertheless, they were to have been expected with certainty to take place from the irritation produced by the depressed portions of bone on the membranes and substance of the brain, if allowed to remain so situated. Hence it might have been fairly argued, that these depressed portions of bone should have been immediately elevated, and removed from the brain. In such cases, it is too late to attempt this after symptoms of inflammation or effusion have taken place, as the danger cannot then be averted: and, accordingly, as I have shewn above, it is an established principle in surgery to elevate or remove depressed fragments of bone from the surface of the brain, in all cases of compound fracture of the skull with depression, whether symptoms appearing to demand interference have come on or not.

"From the above circumstances in this case, if the person accused had not pleaded guilty of culpable homicide, he would probably have been exculpated on the plea of *malum regimen*. There was no effusion of blood within the cranium, and no concussion of the brain to have proved fatal: hence it might be legitimately inferred that, if the inflammation had been averted by removing the cause of irritation, he very probably would have lived.

"Bell was sentenced to seven years' transportation." (Ib.)

It is well known that the medico-legal examination of wounds is a matter of serious importance, and that much assistance may be often rendered to the course of justice by determining the nature of the weapon or instrument with which a wound on a deceased person has been inflicted. This can only be done by a close anatomical inspection of the injury, assisted by a cautious judgment. Several cases reported in the volume prove that the author possesses that tact and cool reflection which, in moments of difficulty are so necessary to a medical jurist; and without which he must sometimes be exposed to the risk of leading to the condemnation of an innocent person, or of ruining his professional character. Among the cases related by Mr. Watson is that of Mrs. Calderhead, (C. 207,) who had died from excessive hemorrhage, in consequence of a wound which she had received on the left labium pudendi. As the reasoning respecting the means by which this wound had been caused is given in our author's best style, we shall here quote it. It may serve as a useful model for medico-legal reports. Externally, the wound consisted of a clean incision, about three-quarters of an inch in length, having a straight direction, parallel with the margin of the labium. A finger, when introduced, entered into a bloody cavity, as large as a small hen's egg, from which it might be passed to a greater depth in three different directions,—viz. upwards, towards the under part of the symphysis pubis; downwards, towards the perineum; and backwards, by the side of the vagina and rectum. Its greatest depth was between two and three inches. Now, it appears that some pieces of a broken wine-glass had been found adjoining some of the blood, on the spot where the deceased had received the wound; and it became, therefore, important to determine whether or not the wound could have been occasioned by her having fallen upon these broken pieces of glass. The following is Mr. Watson's remarks on this point:

"1. Let us consider, Is it physically possible that any of the portions of the broken wine-glass, or of any wine-glass in common use, could have made such a wound? A portion of glass, capable of forming such a wound, must have been between two and three inches long, about three-fourths of an inch broad, having a sharp cutting edge, and some degree of point; and it must also have possessed sufficient strength to permit its having been moved about and thrust in different directions, without breaking. To form such a piece out of a common wine-glass, is evidently beyond the utmost ingenuity of man; far less, then, could such a portion have been formed by accidental fracture.

"The broken portion of glass found consisted of the stalk of a common wine-glass, without the foot, having at its upper part the bottom of the cup of the glass, of about an inch in diameter, attached to it transversely. Nearly the whole of the cup was broken off, leaving only a few fragments of the sides, projecting upwards from the bottom which remained. The stalk was about an inch or an inch and a half in length, and its lower part had been clean broken across, leaving no sharp point. It was, therefore, quite obvious that this fragment of glass was completely incapable of forming the wound in question; for the length, form, and sharpness which were requisite were completely wanting, and any wound which could have been occasioned by it must have been quite different in its character. A wound from such a piece of broken glass would have been a lacerated wound, not a clean incision; not larger internally than externally, and could not have had several different directions internally. The upper end of the glass would have made several small lacerated wounds, while the lower end could not have made any wound at all. I, therefore, came to the conclusion that it was physically impossible for the wound in question to have been produced by any portion of a broken wine-glass such as that found." (P. 260.)

In the remarks on Gun-shot Wounds a curious instance is mentioned, in which the exact spot from which a gun was fired was determined by observing two fixed points where the bullets had struck. It occurred at Ayr, in 1831.

"Several shots had been fired into the church through a window. The bullets left holes in the window, and left marks where they had struck, at the opposite side of the church. By these, the exact place (the window of a house on the opposite side of the street,) from whence the shots had been fired was ascertained by mathematical observation." (P. 264.)

Chapter XI. is devoted to a slight sketch of the subject of Infanticide. We cannot say much for the manner in which the author has stated it. There is no attempt at original observation, and, on the whole, it may be considered as a condensed abstract of the essay of Beck on the same subject. Some passages are open to criticism. In examining the question, " Was the child born dead or alive?" we find the following among the "tests" by which it is said "this question is to be determined." (P. 287.)

"If the child died in the womb, it must have happened from five to twenty days previously to delivery, and, consequently, putrefaction must have advanced to a considerable extent." (Ib.)

This statement appears to us to be opposed to numerous well-ascertained facts. May it not happen that a child, which has died in the womb, may be expelled before the *fifth* day? and is it a necessary consequence that, in all children which have died in the womb, and been expelled after five or six days, putrefaction should have advanced to a considerable extent? We must refer the author to Divergie, and those continental writers whose works he has omitted to consult, for answers to these questions. We suspect, upon reference to the experience of others, he would be inclined to modify his statement. The average weight of the fœtal lungs is described to be about *two ounces;* of the lungs after respiration, about *four ounces;* both of which estimates we believe to be much exaggerated. The remarks on the hydrostatic test are so imperfect, that we cannot suppose them to have been founded on actual observation. The author says, "If the lungs have respired, they are capable of floating the heart and thymus gland along with them;"—again: "If it (a lung) floats, respiration will be conclusive. Nor is this liable to fallacy; for the air which the lung may contain cannot be so completely pressed out by moderate pressure as to prevent the lung from floating, if it had breathed." (Pp. 290, 291.) We here find no account taken of feeble or imperfect respiration; and rules are laid down, as not liable to fallacy, which, if literally followed, cannot but lead to error. But how are we to define what is to be understood by the term "moderate pressure?" It is impossible that such vague directions as these can render the least assistance to a medical jurist; they must rather tend to make him distrustful of all medico-legal rules on a subject of such vital importance. There are several other passages which we had marked out for comment, but we omit them in the hope that, when a second edition of the work appears, the author will set himself the task of examining more fully what has been done by his contemporaries in this branch of science.

The volume concludes with a short essay on Insanity as a plea of exemption from criminal responsibility, which our limits will not permit us to notice. We consider the author's observations respecting Moral Insanity to be highly judicious; and we join him in the wish that this subject may call forth the attention of our high legal authorities, and lead to some amelioration in the law, which we are satisfied, as it at present stands, must occasionally operate with cruelty and inhumanity towards a very wretched class of beings.

We must here conclude our notice of Mr. Watson's work. We have not hesitated to point out what we regard as erroneous in it. This task, we hope, the author will consider we have executed with fairness and candour. If, in ordinary works, this duty must be performed to guard the public from being misled by their titles or pretensions, it becomes much more necessary in the case of a volume like the present, which, we are assured, will deservedly become one of reference and authority. Our readers may rest satisfied that there is much in Mr. Watson's treatise to repay a careful perusal; and, indeed, to be fully appreciated, it must be studied, not merely read. The chapters on Wounds, which constitute four-fifths of the volume, embrace every variety of subject and illustration connected with local injuries; and for this alone the treatise deserves to take the foremost rank in British medico-legal literature. It is not only adapted for the medical practitioner, but it is equally fitted to instruct the coroner, the barrister, and the judge.

---

